# Assessing the healthfulness of pre-packaged beverages and investigating sugar thresholds for revising front-of-pack labels: a case study in Thailand

**DOI:** 10.3389/fnut.2025.1564216

**Published:** 2025-06-02

**Authors:** Hung Nguyen Ngoc, Mayuree Ditmetharoj, Nipa Rojroongwasinkul, Pattanee Winichagoon, Wantanee Kriengsinyos

**Affiliations:** ^1^Doctor of Philosophy Program in Nutrition, Faculty of Medicine Ramathibodi Hospital and Institute of Nutrition, Mahidol University, Nakhon Pathom, Thailand; ^2^Food and Drug Administration, Ministry of Public Health, Nonthaburi, Thailand; ^3^Food and Nutrition Academic and Research Cluster, Institute of Nutrition, Mahidol University, Nakhon Pathom, Thailand

**Keywords:** beverage composition, sugar-sweetened beverages, front-of-package nutritional labeling, sugar threshold, non-nutritive sweeteners, nutrition policy, Thai food supply

## Abstract

Thailand's implementation of the “Healthier Choice” logo (THCL) as a front-of-pack nutritional labeling (FOPNL) scheme aims to promote healthier consumer food choices. In response, the beverage industry has increasingly replaced nutritive sweeteners (NS) with non-nutritive sweeteners (NNS) in their products. This study evaluates the energy and sugar content, sweetener usage, and overall healthfulness of pre-packaged beverages in Thai supermarkets, while also assessing their compliance with WHO “free sugar” consumption guidelines and exploring appropriate sugar thresholds for revising the THCL criteria. A cross-sectional audit was conducted in a major Bangkok supermarket between March and April 2022, collecting data on 881 pre-packaged beverages. To ensure a comprehensive evaluation, these beverages were further categorized based on product ingredients, THCL status, and sweetener type. Healthfulness was assessed using three validated nutrient profiling systems (NPS), and sugar threshold scenarios were evaluated using receiver operating characteristic analysis and area under the curve metrics. The median energy and sugar content per 100 ml were 37.9 kcal (21.9–52.2) and 5.00 g (2.0–7.8), respectively. Overall, pre-packaged beverages in Thai supermarkets were predominantly classified as “less healthy,” with 40.1% containing at least one NNS. Products carrying the THCL logo (27.7%) had lower energy and sugar content, indicating a “healthier” profile. However, these beverages also exhibited a higher prevalence of NNS compared to unlabeled products. Regardless of beverage type, consuming a typical bottle containing NS or a combination of NS and NNS in a single sitting often exceeded the WHO's recommended daily limit of 5% of total energy intake from free sugars. Thus, the study also proposes a phased approach to sugar reduction, initially lowering the sugar threshold to 5.0 g/100 ml, followed by a further reduction to 4.0 g/100 ml. In conclusion, the study underscores the widespread use of NNS in Thai pre-packaged beverages and identifies an overall “less healthy” nutritional profile across product categories. Reducing sugar content and implementing incremental sugar benchmarks in the THCL scheme are essential steps toward improving the healthfulness of pre-packaged beverages in Thailand and aligning these products more closely with WHO guidelines on sugar consumption.

## Introduction

Unhealthy dietary patterns are a critical global public health concern, significantly contributing to rising rates of obesity and non-communicable diseases (NCDs) (1). Among the key contributors to this health crisis, sugar-sweetened beverages (SSBs), have been closely examined for their role in promoting unhealthy diets. Extensive reviews consistently reveal that SSB consumption, a major source of dietary sugar, is associated with weight gain, higher obesity rates, and an increased risk of metabolic disorders such as type 2 diabetes mellitus, cardiovascular disease, and certain cancers (2–6). Therefore, reducing SSB consumption has become a major goal of public health initiatives. Among the various policy strategies aimed at reducing sugar consumption, including marketing restrictions (7), healthy school policies (8), and sugar taxes (9), the implementation of front-of-pack nutrition labels (FOPNLs) is particularly prominent. The World Health Organization (WHO) recommends the use of simplified FOPNLs on packaged foods and beverages as an evidence-based and cost-effective means to empower consumers to make healthier choices and to encourage the food industry to produce healthier products (10–12). However, despite the global adoption of FOPNLs, there is no international consensus on specific nutrient benchmarks (such as sugar and/or artificial sweeteners) to optimize their effectiveness in promoting healthier diets.

In Thailand, there has been a significant and concerning rise in sugar and sweetener consumption over the years. Between 1983 and 2009, the average annual per capita intake increased from 12.7 to 31.2 kg (13), and this figure continued to climb, reaching 51.95 kg in 2022 (14). On average, Thai individuals consume ~25 teaspoons or 100 g of sugar per day (15), which is four times higher than the WHO recommended limit (16). The primary source of this excessive sugar intake is sugar-sweetened beverages (SSBs) (17). The widespread availability (18), affordability (19), and diverse variety of SSB products (20) have significantly contributed to the overconsumption of these beverages, exacerbating the issue.

The “Thailand Healthier Choice Nutritional Labelling” (THCL) scheme, introduced voluntarily by the government in 2016, plays a pivotal role in addressing the issue of unhealthy diets (21). Its primary aim is to assist consumers in making healthier food choices by highlighting healthier options (e.g., low energy, sugar, and fat) within each food category through a criteria-based nutrition label (21, 22). Over the past 5 years, the THCL scheme has shown promising progress in encouraging the reformulation of food products, particularly in the realm of pre-package beverages (23, 24). Many food manufacturers in the Thai market have launched their healthier beverages options and replaced nutritive sweetener (NS) with non-nutritive sweeteners (NNS), resulting in a wide variety of beverages sweetened exclusively with NNS or a combination of NS and NNS (25, 26). Despite the increasing trend of using NNSs in beverages, few studies have examined the sugar content and sweetener usage in beverages within the Thai market. Therefore, the main objectives of this study were to evaluate the nutritional composition and use of sweeteners in pre-packaged beverages in Thai supermarkets, assess the healthfulness profile using various nutrient profiling systems (NPSs), and identify appropriate total sugar thresholds for distinguishing the healthfulness of beverages to improve the criteria of the FOPNL scheme in this country.

## Materials and methods

### Study design and setting

A cross-sectional supermarket audit was carried out for a duration of 2 weeks in March to April 2022 at one of Thailand's major supermarket chains in Bangkok Metropolitan Region, Thailand. This particular supermarket chain holds a substantial number of outlets which accounts of ~25.3% across the country (27).

### Data collection and management

To collect data on product packaging, investigators utilized smartphones to capture various information such as price, front-of-pack labels, ingredient lists, nutrition information panel (NIP), health and nutrition claims, as well as any additional logos and endorsements. In cases where a product was available in multiple packaging sizes, only the most popular size was recorded to prevent redundancy in the data collection process. Data from photographs was then extracted into a Microsoft Excel spreadsheet (Redmond, WA, US) for further analysis.

The data entry process involved collecting essential information from the NIP for both per serve and per 100 g or ml. This included energy (kcal), total fat (g), saturated fat (g), protein (g), total carbohydrate (g), fiber (g), total sugar (g), and sodium (mg). In Thailand, it is not mandatory to provide specific details about fruits, vegetables, pulses, nuts, rapeseed, walnut, and olive oils (FVPNRWO) (%) or fruit, vegetable, nut, and legume (FVNL) (%) on the NIP. Therefore, appropriate levels were estimated using various sources such as the ingredients list, generic food composition databases, or by comparing similar products using established methods. The estimation of FVPNRWO and FVNL content was conducted in two steps according to recommendations from previously published approaches (28) and is detailed in Supplementary Figure 1.

**Figure 1:**
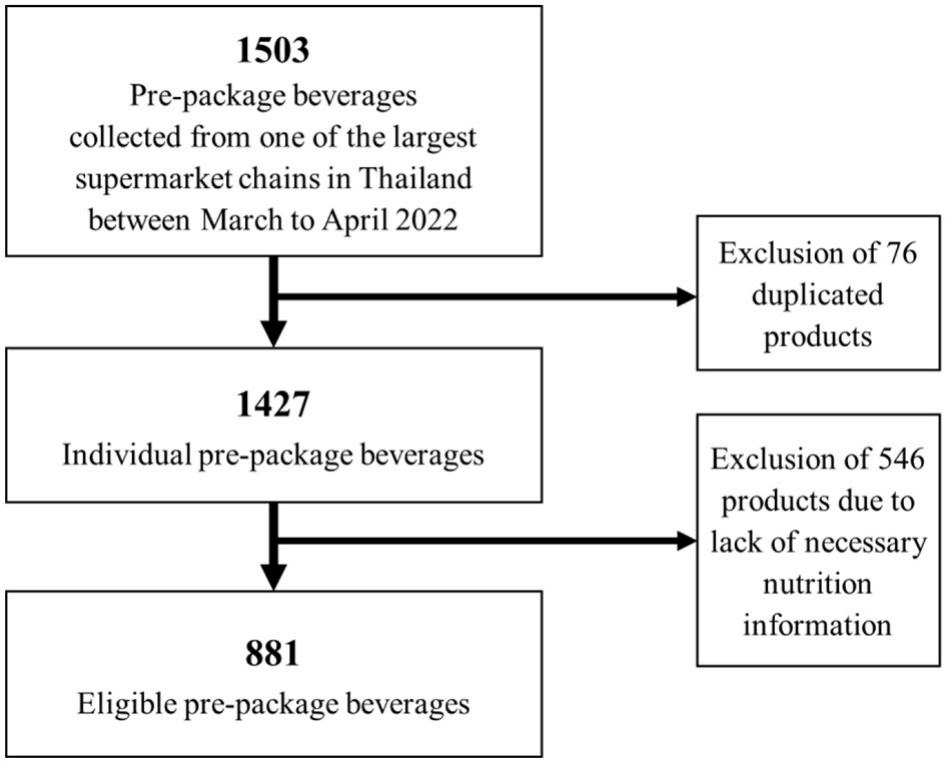
Flowchart of the eligible number of pre-packaged beverages included in this analysis.

To ensure the precision and reliability of the data, a comprehensive review was conducted by a second researcher, meticulously scrutinizing for any discrepancies or errors. Trained nutrition researchers were responsible for the subsequent data cleaning and analysis process. Duplicate products were identified and addressed accordingly. For all products, the NIP data related to the products, as prepared according to the manufacturer's instructions, were utilized. If such data were unavailable, the products were excluded from the analysis (Figure 1). Rigorous checks were performed to verify accuracy by comparing the maximum and minimum nutritional values against the pack images. NIP data presented as “per serving” were standardized to either per 100 ml or 100 g, depending on the product form. To maintain consistency and facilitate comparisons, energy values presented on the product packaging, originally expressed in kilocalories, were converted to kilojoules using the internationally recognized conversion factor of 4.184. Similarly, sodium values, initially indicated in milligrams, were converted to salt in grams using a conversion factor of 0.0025. These conversions ensure standardized units of measurement for accurate analysis and interpretation of the data (29). Values indicated as “less than” on the NIP were adjusted to the nearest whole number for analysis purposes. For instance, if the sugar content was listed as <1.0 g/100 g, it was adjusted to 0.5 g/100 g.

### Definition of pre-packaged beverage

Adapted from the Codex Alimentarius (30), pre-packaged beverages are defined as drinks that are packaged in containers and ready for direct sale to consumers. These beverages are typically sealed and labeled before being sold, ensuring convenience and eliminating the need for additional preparation. They are available in a wide variety of forms and packaging formats, including liquid forms (e.g., bottled or canned ready-to-drink beverages) and solid forms in a discontinuous state (e.g., instant powder beverages in cartons or packets).

In this study, to ensure a systematic assessment of beverage healthfulness, we evaluated all beverages that meet the above packaging criteria (both liquid and solid forms). For liquid-form beverages, the nutritional analysis was conducted directly using the NIP provided on the packaging. For solid-form beverages, such as malted chocolate, cocoa powder, coffee powder, or tea powder, the nutritional analysis was based on the values stated per 100 ml of the prepared beverage (after dilution with water according to the manufacturer's instructions), rather than per 100 g/ml as sold. This approach ensures consistency and accuracy in comparing the nutritional profiles of beverages across different forms and packaging types, allowing for a fair and standardized evaluation of their healthfulness.

### Pre-packaged beverage categorization

In order to allow for a comprehensive analysis of the beverages' nutritional profiles, healthfulness, and sweetener content, this study classified pre-packaged beverages into three categories: (i) based on ingredient composition, (ii) based on THCL status, and (iii) based on sweetener use.

### Categorization based on ingredients

The World Health Organization (WHO) broadly defines sugary beverages as all drinks containing free sugars, a category that includes a wide array of carbonated and non-carbonated drinks, juices, concentrates, flavored water, energy and sports drinks, ready-to-drink tea and coffee, and flavored milk beverages (31). While the WHO's definition does not include plant-based milk substitutes, even though they can contain considerable amounts of free sugars (32), our study adopted a targeted classification of pre-packaged beverages into nine distinct types (detailed alphabetically in Supplementary Table 1): (i) Carbonated drinks, (ii) Coffee, (iii) Energy drinks, (iv) Flavored water, (v) Malted, chocolate, and cocoa (MCC) drinks, (vi) Non-100% fruit and/or vegetable juice (Non-100% FVJ) drinks, (vii) Plant-based milk substitutes, (viii) Sports drinks, and (ix) Tea. This strategic categorization, which included plant-based milk substitutes while excluding flavored milk, was driven by several key considerations relevant to our study's context in Thailand. Firstly, the Food and Drug Administration of Thailand (Thai FDA), the local regulatory authority, classifies flavored milk as a dairy product—a processed form of cow's milk with added flavorings (33, 34). This classification is based on flavored milk's distinct nutritional profile, characterized by significant levels of milk protein, milk fat, and calcium, differentiating it from general beverages (35). Secondly, our analysis of calcium content, a key nutrient associated with dairy, indicated that most flavored milk products in our database met the calcium criteria for dairy beverages under the HSR system (specifically Group 1D). In contrast, plant-based milk substitutes predominantly fell below this calcium threshold, typically falling within general beverage groups like Group 1 (36). Finally, one of our objectives of exploring potential revisions to the FOPNL scheme's sugar thresholds also informed our classification, as it aligns with the existing THCL criteria. Specifically, plant-based milk substitutes, categorized within the beverage group under the THCL, currently share a consistent sugar threshold of 6.0 g/100 ml with other beverages included in our analysis. Conversely, flavored milk, classified as a dairy product by the THCL, operates under a sugar threshold of 8.0 g/100 ml (21). Therefore, while acknowledging the WHO broad definition of sugary beverages, our study adopted a focused classification tailored to the Thai regulatory landscape and our specific research objectives. By strategically excluding flavored milk and including plant-based milk substitutes, we aimed to analyze a more regulatory coherent group of beverages with similar sugar thresholds, ultimately enhancing the relevance and applicability of our findings for informing targeted sugar reduction strategies in Thailand (37).

#### Categorization based on THCL status

Since the THCL scheme was introduced in 2016 (23), pre-packaged beverages in the Thai market are now available either with or without the THCL label. For the purpose of this study, we classified beverages based on THCL status into two groups: (i) Labeled with the THCL logo and (ii) Not labeled with the THCL logo.

#### Categorization based on sweetener use

Pre-packaged beverages are divided into four groups according to the two types of sweeteners (nutritive sweeteners—NS and non-nutritive sweeteners—NNS), as defined in Supplementary Table 2: (i) Only added sugar or NS beverages (containing only added sugar or more listed nutritive sweeteners), (ii) Only NNS beverages (containing one or more listed non-nutritive sweeteners), (iii) Mix NS + NNS beverages or (containing both types of sweeteners), and (iv) Unsweetened beverages (containing no added sugar or sweeteners in the ingredient list).

### Assessment of the healthfulness of beverages

NPS use algorithms to evaluate the nutritional quality of food and beverage products based on the presence or amounts of specific nutrients (38). With hundreds of such systems developed, selecting the most appropriate one can be challenging. In this study, we chose NPSs based on their validated evidence (i.e., content, convergent, and criterion validity) to ensure their accuracy in assessing the healthfulness of foods and beverages (39, 40). Previous research has demonstrated a correlation between consuming foods rated as healthier by these NPSs and improved health outcomes (40–47). Specifically, we evaluated the healthfulness of pre-packaged beverages using the updated 2023 Nutri-Score algorithm, Health Star Rating (HSR), and Chilean Warning Label (CWL), ensuring a robust and evidence-based assessment of their nutritional quality.

#### Calculation of the Nutri-Score grade

Nutri-Score is a front-of-pack nutrition label that uses a five-letter scale (A to E) to indicate the nutritional quality of food and beverage products. Developed by Santé Publique France, it is widely adopted across European Union (EU) countries. In this study, we evaluated the healthfulness of beverages using the latest version of the Nutri-Score algorithm (v2.2023) (48, 49). This algorithm calculates a score based on the modified Food Standard Agency Nutrient Profiling System (FSAm-NPS), which incorporates sweeteners into the model and assesses the nutritional composition per 100 ml of the beverage. All items in this study were categorized under the beverages group. The score is determined by subtracting points for “negative nutrients” (e.g., sugar, sodium, saturated fat) from points for “positive nutrients” (e.g., protein, fiber, fruits, vegetables, and nuts). Scores range from −15 (healthiest) to +40 (least healthy), with detailed calculating and grading criteria provided in Supplementary Table 3.

#### Calculation of the Health Star Rating grade

The HSR system, implemented in Australia and New Zealand, evaluates the healthfulness of products by assigning them a rating from 0.5 to 5.0 stars based on their nutritional composition. In this study, we calculated the HSR using the “Guide for Industry to the HSR Calculator,” version V8.2023 (36). Similar to Nutri-Score, the HSR algorithm is a modified version of the FSAm-NPS, though it does not incorporate sweeteners into the calculation. All beverages in this study were categorized into either Group 1 or Group 1D, depending on their calcium content (applied only to plant-based milk substitutes and MCC drinks). The HSR was determined by adjusting the “baseline” points for energy and sugar per 100 ml with “modifying” points for the content of FVNL. These points were then converted into the HSR using a scoring matrix. The calculation process and color grading are detailed in Supplementary Table 3.

#### Calculation of the Chilean Warning Label grade

The warning label system, firstly introduced in Chile, targets packaged foods and beverages with high levels of sugar, saturated fats, sodium, and/or calories. Its purpose is to inform consumers about the nutritional quality of these products and promote healthier dietary choices. The CWL, which are black-and-white octagons, use nutritional data from 100 g or ml of the product (50). If a product exceeds the acceptable limits for sugar, sodium, saturated fat, or calories set by the Chilean Ministry of Health, the label displays the phrase “High in”. Each product must display a warning for each nutrient that exceeds these thresholds, meaning products may have up from 0 to 1 and even to four warnings based on their nutritional composition (50). The specific thresholds and graded color system are detailed in Supplementary Table 3.

### Assessment of compliance with WHO “free sugar” guidelines

In this study, the findings of sugar content of beverages were further compared with the WHO guidelines on free sugars to assess their alignment with dietary recommendations. The WHO guidelines provide two threshold recommendations: (1) free sugars should not exceed 10% of total dietary energy (i.e., 50 g), and (2) additional health benefits are achieved when free sugars contribute below 5% of total dietary energy (i.e., 25 g), based on a 2,000 kcal diet (16). To evaluate the potential contribution of free sugars in pre-packaged beverages to daily dietary energy intake, the medians and IQRs of key indicators were calculated for each beverage category, as follows:

(i) The percentage contribution of one serving of a beverage to the WHO free sugar guidelines:


$$\begin{array}{l} \text{ Percent contribution }\left(\text{\%}\right)\\ =\frac{\text{Sugar per servin}{\text{g}}^{*}\;\left(\text{g}\right)}{\text{WHO recommended sugar limit }\left(\text{g}\right)}\times 100\; \end{array}$$

^*^Serving size was calculated as the median value of all beverages within that category, based on the manufacturer-declared serving size information on the NIP.

(ii) Milliliters of a beverage that would need to be consumed to exceed the WHO free sugar guidelines:


$$\begin{array}{l} \text{ Volumne of beverage consumed }\left(\text{ml}\right)\\ =\frac{\text{Volumne of beverage per servin}{\text{g}}^{*}\;\left(\text{ml}\right)}{\text{Percent contribution }\left(\text{\%}\right)}\times 100\; \end{array}$$

(iii) Servings of beverage that would need to be consumed to exceed the WHO free sugar guidelines:


$$\begin{array}{l} \text{ Number of servings}\\ =\frac{\text{WHO recommneded sugar limit }\left(\text{g}\right)}{\text{Sugar per serving }\left(\text{g}\right)\;}\; \end{array}$$

## Statistical analyses

Following data cleaning in Microsoft Excel 365, we performed statistical analyses using the IBM SPSS Statistics for Windows, version 25.0 (IBM Corp., Armonk, NY, US), and the graphs were created using GraphPad Prism version 9.5 (GraphPad Software, San Diego, CA, US). Statistical significance was determined at a *p-*value < 0.05. Descriptive statistics were used to present the medians and IQRs for energy (kcal/100 ml) and total sugar content (g/100 ml), as well as the number and percentage of NS or NNS usage for each beverage category. To compare median and categorical data between groups, Mann Whitney U test and Chi-squared test were employed, respectively. The distribution of healthfulness across different NPSs was determined by calculating the frequencies of beverages within various sub-categories.

Currently, the THCL scheme lists five categories under beverages (23), each with specific thresholds for energy, sugar, sodium, or fat. Despite these variations, all categories share a common sugar threshold of 6 g per 100 ml. Therefore, to assess the effectiveness of the THCL's current sugar threshold in distinguishing between “healthier” and “less healthy” SSBs according to various NPSs, a comparative analysis was conducted using multiple sugar threshold scenarios. To improve the healthfulness of SSBs, a reduction in the sugar threshold was deemed necessary. Consequently, different sugar benchmarks ranging from 2.0 to 5.5 g/100 ml were examined to determine the most reasonable threshold. Receiver Operating Characteristic (ROC) curves were utilized, and the Area Under the Curve (AUC) was calculated. A pairwise comparison of the AUCs for the current sugar threshold and the alternative scenarios was performed using the Hanley and McNeil method (51).

## Results

### Nutritional composition of pre-packaged beverages

Among the final of 881 included beverages, approximately one-third (*n* = 244, 27.7%) displayed THCL logos. The nutrient composition and characteristics of sweetener usage are summarized in Table 1. The median energy and sugar content of pre-packaged beverages in Thai supermarkets were 37.9 kcal and 3.0 g per 100 ml, respectively. There was substantial variability across beverage categories. The highest sugar contents were found in non-100% FVJ drinks (9.0 g/100 ml), while the lowest were in tea (3.0 g/100 ml). Beverages with THCL logos showed significantly lower energy and sugar content compared to those without the THCL logos. The medians and interquartile ranges (IQRs) for energy and sugar level were 22.5 (8.8–44.4) vs. 41.67 (54.8–26.4) kcal/100 ml, and 3.0 (0.1–4.9) vs. 6.0 (3.3–9.0) g/100 ml for THCL and no-THCL, respectively. Category analysis revealed statistically significant differences in energy and sugar content among “the most popular consumed” beverage types, including carbonated drinks, flavored water, non-100% FVJ drinks, coffee, and tea. However, “performance drinks”, such as energy drinks and sports drinks, and “alternative dairy beverages”, such as plant-based milk substitutes and MCC drinks, showed no significant differences.

**Table 1:** Characteristics of pre-packaged beverages across beverage category (*n* = 881).

**Characteristic**	**Overall**	**Labeled with THCL logo**	**Not labeled with THCL logo**	Difference^*^	***p*-value**
**Carbonated drink**					
Number (*n*)	76	21	55		
**Nutrient Composition—Median (IQR)**					
Energy (kcal/100 ml)	20.0 (6.1–30.0)	0.0 (0.0–19.4)	25.0 (18.5–35.0)	25.00	<0.001
Sugar (g/100 ml)	4.6 (0.5–7.5)	0.0 (0.0–4.2)	6.2 (4.5–7.8)	6.18	<0.001
**Use of Sweetener—*****n*** **(%)**					
Unsweetened	1 (1.3)	0 (0)	1 (1.8)	1.8	N/A
Only NS	18 (23.7)	0 (0)	18 (32.7)	32.7	N/A
Only NNS	18 (23.7)	15 (71.4)	3 (5.5)	65.9	<0.001
Mix NS + NNS	39 (51.3)	6 (28.6)	33 (60)	31.4	0.087
**Coffee**					
Number (*n*)	110	30	80		
**Nutrient Composition—Median (IQR)**					
Energy (kcal/100 ml)	44.1 (30.7–59.4)	26.4 (5.2–37.0)	52.1 (39.1–63.1)	25.75	<0.001
Sugar (g/100 ml)	3.9 (0.4–6.8)	3.3 (0.0–4.9)	4.6 (0.4–7.2)	1.40	0.018
**Use of Sweetener—*****n*** **(%)**					
Unsweetened	12 (10.9)	7 (23.3)	5 (6.2)	17.1	0.016
Only NS	45 (40.9)	6 (20.0)	39 (48.8)	28.8	0.036
Only NNS	20 (18.2)	4 (13.3)	16 (20.0)	6.7	0.465
Mix NS + NNS	33 (30.0)	13 (43.4)	20 (25.0)	18.4	0.118
**Energy drink**					
Number (*n*)	14	0	14		
**Nutrient Composition—Median (IQR)**					
Energy (kcal/100 ml)	31.7 (25.6–53.8)	N/A	31.7 (25.6–53.8)	N/A	N/A
Sugar (g/100 ml)	7.5 (5.3–10.7)	N/A	7.5 (5.3–10.7)	N/A	N/A
**Use of Sweetener—*****n*** **(%)**					
Unsweetened	0 (0.0)	N/A	0 (0.0)	N/A	N/A
Only NS	1 (7.1)	N/A	1 (7.1)	N/A	N/A
Only NNS	1 (7.1)	N/A	1 (7.1)	N/A	N/A
Mix NS + NNS	12 (85.8)	N/A	12 (85.8)	N/A	N/A
**Flavored water**					
Number (*n*)	192	58	134		
**Nutrient Composition—Median (IQR)**					
Energy (kcal/100 ml)	25.0 (16.9–42.7)	15.6 (6.8–22.0)	33.3 (24.0–48.0)	17.75	<0.001
Sugar (g/100 ml)	4.7 (1.5–6.2)	2.0 (0.0–4.7)	5.0 (3.3–8.7)	3.00	<0.001
**Use of Sweetener—*****n*** **(%)**					
Unsweetened	14 (9.9)	5 (8.6)	14 (10.4)	1.8	0.925
Only NS	44 (22.9)	9 (15.5)	35 (26.1)	10.6	0.310
Only NNS	49 (25.5)	23 (39.7)	26 (19.4)	20.3	0.002
Mix NS + NNS	80 (41.7)	21 (36.2)	59 (44.0)	7.8	0.837
**MCC drink**					
Number (*n*)	37	4	33		
**Nutrient Composition—Median (IQR)**					
Energy (kcal/100 ml)	52.6 (41.7–63.4)	52.4 (48.3–52.6)	55.8 (36.1–64.7)	3.39	0.750
Sugar (g/100 ml)	5.6 (3.2–7.1)	5.0 (4.7–5.3)	6.3 (2.2–7.4)	1.32	0.525
**Use of Sweetener—n (%)**					
Unsweetened	7 (18.9)	1 (25.0)	6 (18.2)	6.8	0.767
Only NS	23 (62.2)	3 (75.0)	20 (60.6)	14.4	0.730
Only NNS	4 (10.8)	0 (0)	4 (12.1)	12.1	N/A
Mix NS + NNS	3 (8.1)	0 (0)	3 (9.1)	9.1	N/A
**Non-100% FVJ drink**					
Number (*n*)	197	19	178		
**Nutrient Composition—Median (IQR)**					
Energy (kcal/100 ml)	45.0 (36.0–51.0)	22.5 (22.2–22.9)	45.2 (40.0–54.7)	22.73	<0.001
Sugar (g/100 ml)	9.0 (6.1–11.3)	5.0 (4.5–5.6)	9.5 (7.4–11.5)	4.50	<0.001
**Use of Sweetener—n (%)**					
Unsweetened	127 (64.5)	10 (52.6)	117 (65.7)	13.1	0.499
Only NS	38 (19.3)	4 (21.1)	34 (19.1)	2.0	0.854
Only NNS	17 (8.6)	0 (0.0)	17 (9.6)	9.6	N/A
Mix NS + NNS	15 (7.6)	5 (26.3)	10 (5.6)	20.7	0.002
**Plant-based milk substitute**					
Number (*n*)	129	68	61		
**Nutrient Composition—Median (IQR)**					
Energy (kcal/100 ml)	52.2 (40.8–64.0)	52.0 (44.4–59.3)	55.6 (38.9–70.7)	3.54	0.173
Sugar (g/100 ml)	3.3 (1.2–5.0)	3.3 (2.0–4.6)	3.2 (0.6–5.6)	0.13	0.828
**Use of Sweetener—n (%)**					
Unsweetened	35 (27.1)	12 (17.6)	23 (37.7)	20.1	0.029
Only NS	86 (66.7)	48 (70.6)	38 (62.3)	8.3	0.565
Only NNS	2 (1.6)	2 (2.9)	0 (0.0)	2.9	N/A
Mix NS + NNS	6 (4.7)	6 (8.8)	0 (0.0)	8.8	N/A
**Sports drink**					
Number (*n*)	8	1	7		
**Nutrient Composition—Median (IQR)**					
Energy (kcal/100 ml)	24.5 (6.0–30.3)	24.0 (24.0–0.240)	25.0 (0.0–32.0)	1.00	N/A
Sugar (g/100 ml)	5.8 (1.3–6.9)	5.6 (5.6–5.6)	6.0 (0.0–7.2)	0.40	N/A
**Use of Sweetener—n (%)**					
Unsweetened	0 (0.0)	0 (0.0)	0 (0.0)	0	N/A
Only NS	4 (50.0)	1 (100)	3 (42.9)	57.1	0.450
Only NNS	2 (25.0)	0 (0.0)	2 (28.6)	28.6	N/A
Mix NS + NNS	2 (25.0)	0 (0.0)	2 (28.5)	28.5	N/A
**Tea**					
Number (*n*)	118	43	75		
**Nutrient Composition—Median (IQR)**					
Energy (kcal/100 ml)	14.6 (0.0–32.0)	12.0 (0.0–20.0)	25.0 (0.0–40.0)	13.00	0.036
Sugar (g/100 ml)	3.0 (0.0–5.8)	1.6 (0.0–3.7)	4.3 (0.0–7.2)	2.72	0.038
**Use of Sweetener—n (%)**					
Unsweetened	38 (32.2)	10 (23.3)	28 (37.3)	14.0	0.195
Only NS	29 (24.6)	8 (18.6)	21 (28.0)	9.4	0.322
Only NNS	9 (7.6)	5 (11.6)	4 (5.3)	6.3	0.233
Mix NS + NNS	42 (35.6)	20 (46.5)	22 (29.3)	17.2	0.132
**Total beverage**					
Number (*n*)	881	244	637		
**Nutrient Composition—Median (IQR)**					
Energy (kcal/100 ml)	37.9 (21.9–52.2)	22.5 (8.8–44.4)	41.7 (54.8–26.4)	19.17	<0.001
Sugar (g/100 ml)	5.00 (2.0–7.8)	3.0 (0.1–4.9)	6.0 (3.3–9.0)	3.00	<0.001
**Use of Sweetener—n (%)**					
Unsweetened	239 (27.2%)	45 (18.4%)	194 (30.5%)	12.1	0.002
Only NS	288 (32.7%)	79 (32.4%)	209 (32.8%)	0.4	0.920
Only NNS	122 (13.8%)	49 (20.1%)	73 (11.5%)	8.6	0.002
Mix NS + NNS	232 (26.3%)	71 (29.1%)	161 (25.3%)	3.8	0.322
**Average number of sweeteners used**	**0.71**	**0.87**	**0.65**		

IQR, Interquartile range; FVJ, Fruit and/or Vegetable Juice; MCC, Malted Chocolate and Cocoa; NNS, Non-nutritive sweetener; NS, Nutritive Sweetener; N/A, not applicable.

* The differences were compared between pre-packaged beverages labeling with THCL logo and those without labeling. The comparison was conducted using the Mann Whitney U test and Chi-square tests appropriately. N/A stands for “not applicable” indicating that the data or metric was either unavailable or insufficient for comparison, making it impossible to calculate the statistics.

Regarding the use of sweeteners, 40% of pre-packaged beverages (354 out of 881) contained at least one NNS. Among these, 32.7% contained only NNS, with usage ranging from 7.1% to 66.7% across different categories. Notably, beverages carrying the THCL logo (*n* = 120, 49.2%) had a significantly higher proportion of NNS usage compared to those without labeling (*n* = 234, 39.6%; *p* = 0.002). A closer look at different beverage categories revealed that energy drinks had the highest prevalence of NNS usage (*n* = 13, 92.9%), followed by carbonated drinks (*n* = 57, 75%) and flavored water (*n* = 129, 67.2%). In contrast, the lowest proportions were observed in plant-based milk substitutes (*n* = 8, 6.3%), non-100% FVJ drinks (*n* = 32, 16.2%), and MCC drinks (*n* = 7, 18.9%).

### Healthfulness profile of pre-packaged beverages

#### Beverage categorization based on ingredients

**Figure 2:**
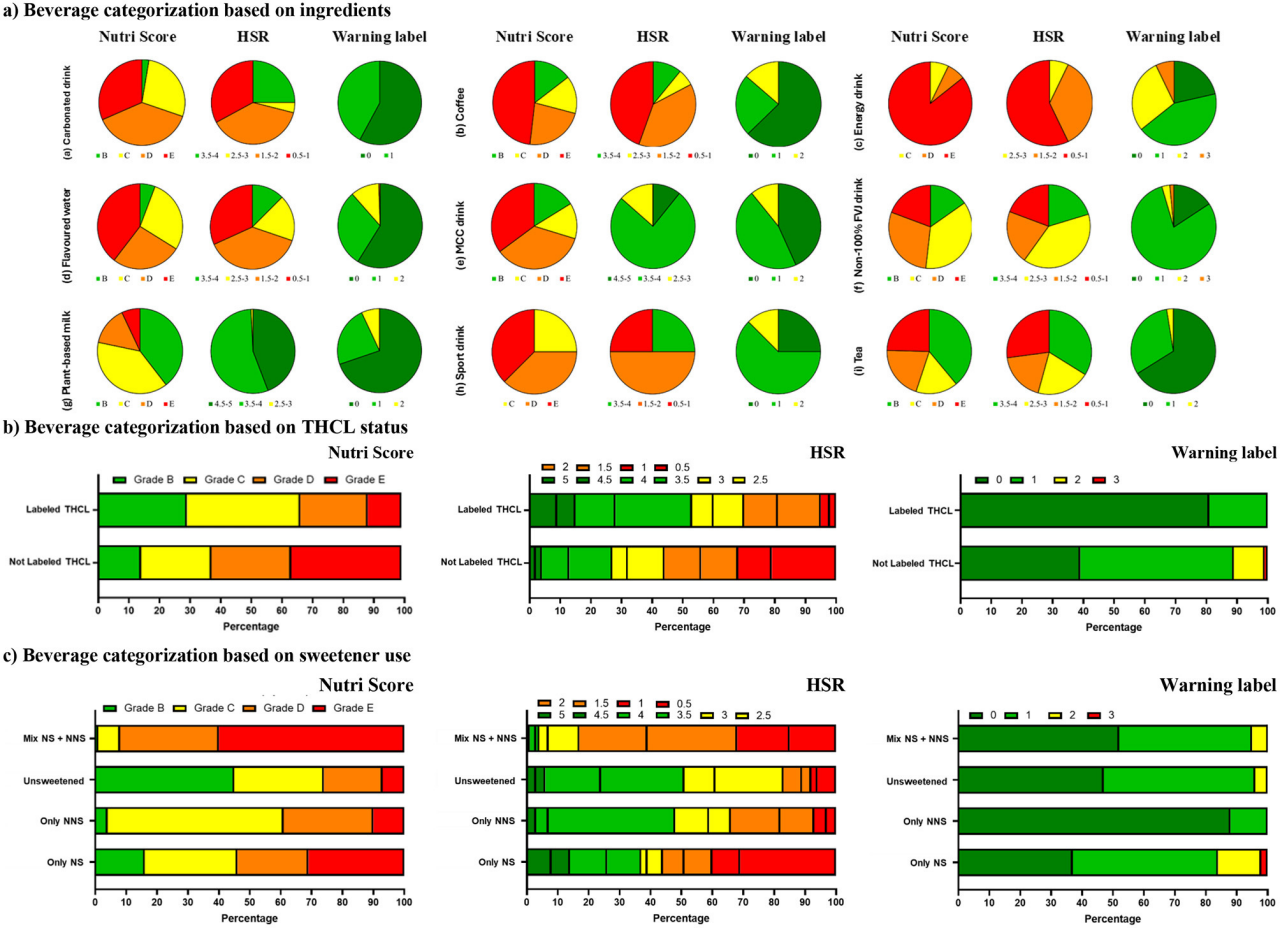
The distribution of healthfulness across pre-packaged beverage categories, **(a)** based on ingredients, **(b)** based on the THCL status, **(c)** based on sweetener use, as assessed by different nutrient profiling systems.

Figure 2a presents the distribution of pre-packaged beverage healthfulness across different categories based on ingredient composition, as assessed by various nutrient profiling systems (NPSs). According to the Nutri-Score classification, more than half of the beverages were categorized as “less healthy,” with grade “E” being the most common (29.2%), followed by grade “C” (27.2%) and grade “D” (25.2%). Meanwhile, the HSR system showed a more balanced distribution, with 44.9% of beverages classified as “healthy” (HSR score ≥ 3.5) and 44.2% as “less healthy” (HSR score < 3.5). The overall median HSR was 2.5 (IQR: 1.5–3.5). In contrast, the CWL system classified 50.6% of beverages as “healthier” which free from warning labels, while 41.7% carried at least one warning label.

A closer examination of beverage categories revealed that the Nutri-Score and HSR systems assigned similar healthfulness ratings to tea, coffee, flavored water, and non-100% FVJ drinks. However, discrepancies emerged in the classification of carbonated drinks, sports drinks, and energy drinks. Notably, plant-based milk substitutes and MCC drinks exhibited significant inconsistencies. Most beverages in these two categories were classified as “healthy” under the HSR system (score ≥ 3.5), whereas the Nutri-Score system rated them as “less healthy,” assigning grades C to E.

#### Beverage categorization based on THCL status

Overall, beverages carrying the THCL logo exhibited significantly better healthfulness across various NPSs compared to those without the label, as shown in Figure 2b. According to the Nutri-Score classification, 29.1% of labeled products received a “healthy” grade (“B”), whereas only 14.3% of unlabeled beverages achieved this rating. Consequently, the proportion of “less healthy” beverages was higher among unlabeled products (85.7%) than labeled ones (70.9%). Specifically, 33.6% of labeled beverages were classified under the lowest healthfulness grades (“D” and “E”), compared to 62.3% of unlabeled products. Grade “C” was more also frequently assigned to labeled beverages (37.3%) than to unlabeled ones (23.4%). Similarly, the HSR system also revealed notable differences in healthfulness between labeled and unlabeled beverages. Over half (52.9%) of labeled beverages received a rating of 3.5 stars or higher, whereas only 26.4% of unlabeled products met this threshold. In contrast, 32.0% of unlabeled beverages were assigned the lowest rating (0.5–1 stars), compared to just 4.5% of labeled ones. The median HSR for labeled beverages was 3.5 (IQR: 2.0–4.0), while unlabeled products had a lower median score of 2.0 (IQR: 1.0–3.5). For the warning label, the CWL system highlighted stark differences. The majority (81%) of beverages with the THCL logo were free from warning labels, whereas only 39% of unlabeled products had no warnings.

#### Beverage categorization based on sweetener use

The healthfulness of beverages varied significantly depending on the type of sweetener used, as shown in Figure 2c. According to the Nutri-Score classification, nearly all beverages containing a mix of NS + NNS (99.1%) were categorized as “less healthy” (grades C, D, and E). Similarly, 95.9% of beverages with only NNS and 83.7% of those with only NS also fell into these lower healthfulness categories. In contrast, 45.2% of unsweetened beverages were classified in the “healthy” band with a grade of B. With equivalent findings, the HSR system further reinforced these differences. Beverages with a mix of NS + NNS had the lowest median HSR score of 1.5 (IQR: 1.0–2.0), with 95.7% rated as “less healthy” (HSR < 3.5). Beverages containing only NNS had a median HSR of 3.0 (IQR: 2.0–3.5), with 52.5% scoring below 3.5 stars. Those with only NS had a median HSR of 2.0 (IQR: 0.5–4.0), with 62.5% classified as “less healthy.” In contrast, unsweetened beverages had the highest median HSR of 3.5 (IQR: 2.5–3.5), with 50.6% receiving an HSR of 3.5 or higher, placing them in the “healthy” category. When assessed using the CWL system, beverages containing a mix of NS + NNS (51.7%) and unsweetened beverages (46.9%) had similar proportions of products free from warning labels. However, beverages with only NNS had the highest proportion of warning-label-free products (87.7%), whereas those with only NS had the lowest (37.7%).

### Assessment of compliance with WHO “free sugar” guidelines

The compliance of beverages with the WHO's free sugars consumption guidelines (16) is summarized in Table 2. Overall, the findings indicate that a single serving of beverages containing only NS or a mix of NS + NNS, as recommended by manufacturers, contributes ~24% and 21% of the WHO's guideline, respectively. Based on the median serving sizes specified by manufacturers, the number of servings required to exceed this guideline was 4.2 for beverages with only NS and 4.6 for those with a mix of NS + NNS. When applying the stricter recommendation of limiting free sugars to <5% of total daily energy intake, a single serving of NS-only beverages accounted for 48% of the maximum daily intake, while mixed NS + NNS beverages contributed 42%. To surpass this stricter threshold, the required serving sizes were 2.1 and 2.3, respectively.

**Table 2:** The median and interquartile range of the percentage contribution of pre-packaged beverage containing only NS and those containing mix NS + NNS to meeting/exceeding the WHO's daily recommended limits of sugars intake^*^ (16).

**Adherence to WHO free sugar guidelines**	**Beverage category**	**Only NS Beverage**						**Mix NS** + **NNS Beverage**					
		**% Contribution to WHO guideline per serving^†^**		**Milliliter of beverage consumed to exceed WHO guideline**		**Servings taken to exceed WHO guideline**		**% Contribution to WHO guideline per serving^†^**		**Milliliter of beverage consumed to exceed WHO guideline**		**Servings taken to exceed WHO guideline**	
		**Median**	**IQR**	**Median**	**IQR**	**Median**	**IQR**	**Median**	**IQR**	**Median**	**IQR**	**Median**	**IQR**
Contribution to WHO guideline of 10% dietary energy from free sugars	Carbonated drink	36.0	28.5–43.0	625.0	478.5–751.6	2.8	2.3–3.3	30.0	24.0–30.0	1000.0	694.4–1111.1	3.3	3.3–4.2
	Coffee	20.0	16.0–28.0	740.0	616.5–1208.3	4.8	3.6–6.1	14.0	10.0–19.0	1041.7	821.0–1421.0	7.1	5.0–10.0
	Energy drink	56.0	N/A	303.6	N/A	1.8	N/A	21.0	16.5–35.5	669.6	504.5–911.5	4.9	2.9–6.1
	Flavored water	18.0	10.0–31.0	778.7	527.7–1133.5	5.6	3.2–10.0	18.0	12.0–29.5	1000.0	888.9–1250.0	5.6	3.3–7.7
	MCC drink	30.0	24.0–34.0	770.8	646.2–958.3	3.3	2.9–4.2	24.0	12.0–30.0	733.3	666.7–1350.0	4.2	3.3–4.2
	Non-100% FVJ drink	35.0	26.0–46.5	491.9	396.2–669.1	2.9	2.2–3.8	24.0	20.0–32.0	900.0	692.3–1000.0	4.2	3.1–5.0
	Plant-based milk	17.0	10.0–24.0	1277.8	921.9–1950.0	5.6	4.2–10.0	15.0	11.0–19.5	1267.7	1021.5–2265.6	6.7	5.2–9.4
	Sports drink	26.0	24.0–53.5	833.3	510.8–878.0	3.9	2.1–4.2	31.0	26.0–36.0	828.0	694.4–961.5	3.3	2.8–3.9
	Tea	32.0	9.0–40.0	833.3	652.1–1692.3	2.9	2.5–7.1	27.0	15.5–34.0	1029.4	717.4–1510.4	3.6	2.9–6.3
	**Total**	**24.0**	**14.0–34.0**	**833.3**	**581.7–1254.2**	**4.2**	**2.9–7.1**	**21.0**	**14.0–30.0**	**1000.0**	**745.8–1180.8**	**4.6**	**3.3–7.1**
Contribution to WHO guideline of 5% dietary energy from free sugars	Carbonated drink	72.0	57.0–86.0	312.5	239.2–375.8	1.4	1.1–1.7	60.0	48.0–60.0	500.0	347.2–555.6	1.7	1.7–2.1
	Coffee	40.0	32.0–56.0	370.0	308.2–604.2	2.4	1.9–3.0	28.0	20.0–38.0	520.8	410.5–710.5	3.6	2.5–5.0
	Energy drink	112.0	N/A	151.8	N/A	0.9	N/A	42.0	33.0–71.0	334.8	252.2–455.7	2.4	1.4–3.0
	Flavored water	36.0	20.0–62.0	389.4	263.8–566.8	2.8	1.6–5.0	36.0	24.0–59.0	500.0	444.4–625.0	2.8	1.7–3.9
	MCC drink	60.0	48.0–68.0	385.4	323.1–479.2	1.7	1.5–2.1	48.0	24.0–60.0	366.7	333.3–675.0	2.1	1.7–4.2
	Non-100% FVJ drink	70.0	52.0–93.0	246.0	198.1–334.6	1.4	1.1–1.9	48.0	40.0–64.0	450.0	346.2–500.0	2.1	1.6–2.5
	Plant-based milk	34.0	20.0–48.0	638.9	460.9–975.0	2.8	2.1–5.0	30.0	22.0–39.0	633.9	510.8–1132.8	3.3	2.6–4.7
	Sports drink	52.0	48.0–107.0	416.7	255.4–439.0	1.9	1.1–2.1	62.0	52.0–72.0	414.0	347.2–480.8	1.7	1.4–1.9
	Tea	64.0	18.0–80.0	416.7	326.1–846.2	1.5	1.3–3.6	54.0	31.0–68.0	514.7	358.7–755.2	1.8	1.5–3.2
	**Total**	**48.0**	**28.0–68.0**	**416.7**	**290.9–627.1**	**2.1**	**1.5–3.6**	**42.0**	**28.0–60.0**	**500.0**	**372.9–590.4**	**2.3**	**1.7–3.6**

IQR, Interquartile range; FVJ, Fruit and/or Vegetable Juice; MCC, Malted Chocolate and Cocoa; NNS, Non-nutritive sweetener; NS, Nutritive Sweetener; N/A, not applicable.

* The calculation of percent contribution to WHO free-sugar guidelines was based on a daily intake of 2,000 kcal (16).

† Sugar per serving and serving sizes are calculated and displayed in Supplementary Table 4.

### Sugar-threshold for revision of FOPNL scheme in Thailand

**Figure 3:**
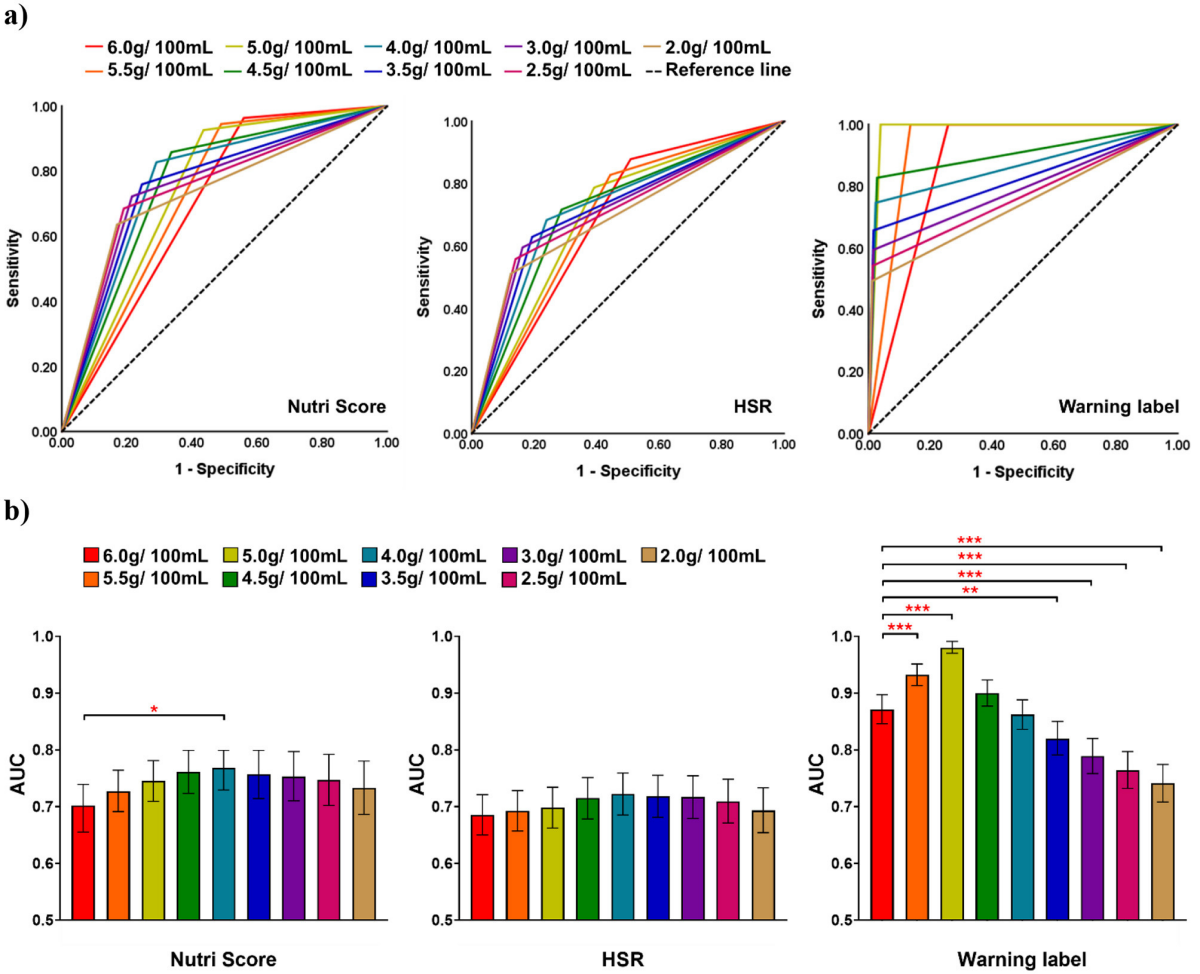
Results of ROC analysis to discriminate between “healthier” and “less healthy” pre-packaged beverages used in the current total sugar threshold of THCL criteria (i.e., 6.0 g/100 ml) and different sugar threshold scenarios (range from 2.0 to 5.5 g/100 ml). **(a)** ROC curve for each NPS. **(b)** AUC value for each NPS. Statistical test results obtained using the Hanley and McNeil method for two correlated ROC curves are also shown (*p < 0.05; **p < 0.01; ***p < 0.001).

The results of the ROC-AUC analysis comparing the current sugar threshold (i.e., 6.0 g/100 ml) with various alternative sugar scenarios (ranging from 2.0 g/100 ml to 5.5 g/100 ml) for distinguishing between “healthier” and “less healthy” beverages using different validated NPSs, are shown in Figure 3. Evaluation measures for each NPS are presented in Supplementary Table 5. The ROC curves for each scenario are overlaid for each NPS (Figure 3a). The AUC with a 95% CI for the current sugar threshold of the THCL scheme (6.0 g/100 ml) were 0.702 (Nutri-Score), 0.685 (HSR), and 0.871 (Warning label). As the sugar benchmark was reduced by 0.5 g units, the AUC gradually increased to its highest point, then decreased, as shown in Figure 3b. By reducing the sugar threshold to 4.0 g/100 ml, the AUC significantly increased by 9.4% for Nutri-Score and 5.4% for HSR, reaching their highest AUC values of 0.768 and 0.722, respectively. On the other hand, for the CWL, the sugar threshold of 5.0 g/100 ml achieved the highest AUC of 0.980, representing a 12.5% increase compared to the current threshold of 6.0 g/100 ml. While the AUC value began to decrease with stricter thresholds, the AUC value at the 4.0 g/100 ml threshold for the CWL still remained high at 0.862, which was not significantly lower than the current threshold.

## Discussion

The present study provides a pioneering and comprehensive examination of the nutritional quality of pre-packaged beverages available in Thai supermarkets. It analyzed key factors such as energy and sugar content, sweetener use, and the volume and serving sizes that exceed the WHO's free sugar consumption guidelines. In an effort to enhance measures for obesity and NCDs prevention, the study also aimed to identify an appropriate sugar threshold for revising Thailand's FOPNL scheme. The findings offer valuable insights to support efforts to improve the healthfulness of beverages in the Thai market, contributing to better public health outcomes.

### What are the energy and sugar levels in pre-packaged beverages available in Thai supermarkets?

Thailand has implemented several measures to address obesity and NCDs, including education campaigns (e.g., the Fatless Belly Thais program), advertising restrictions (e.g., the Soda Ban in Schools), the introduction of an SSB excise tax in 2017, and the FOPNL, under THCL scheme in 2016. Data collected five years after the implementation of these policies revealed that the median and mean sugar content of pre-packaged beverages in Thai supermarkets were 3.0 and 6.2 g per 100 ml, respectively. These figures are notably lower than those in countries without both an SSB tax and FOPNL scheme, such as the US (7.4 g per 100 ml), Taiwan (9.2 g per 100 ml), and Canada (9.6 g per 100 ml) (52, 53). They are also lower than in countries with an FOPNL system but no sugary drink tax, such as Singapore (mean of 6.4 g per 100 ml), Australia (7.0 g per 100 ml), and New Zealand (7.5 g per 100 ml) (53–55). While the causal relationship between these policies and reduced sugar content is not definitively established, the findings might suggest that the combination of fiscal measures (via SSB tax) and informational tools (via FOPNL scheme) may be more effective in reducing sugar content in beverages than either measure alone (56). The SSB tax likely incentivizes manufacturers to reformulate products to lower sugar levels, while the FOPNL scheme empowers consumers to make healthier choices by providing clear nutritional information. Together, these policies may create a synergistic effect, driving both supply-side and demand-side changes in the beverage market (57, 58). However, the evidence for this synergy is not yet strongly conclusive. Therefore, there is a need for further research to compare the relative effectiveness of different taxation strategies and FOPNL formats, as well as to explore how these policy measures interact when applied in combination.

### How prevalent is the use of sweeteners in pre-packaged beverages?

The sugar content of beverages is closely linked to the use of sweeteners, as manufacturers often incorporate NNS to reduce calorie content while maintaining sweetness or combine them with NS to balance taste and health considerations (26). To better understand how these beverages achieve their sweetness, our findings indicate that 40% of pre-packaged beverages contained NNS, either exclusively or in combination with NS, a trend consistent with global patterns. In emerging markets such as Singapore, Hong Kong, and Mexico (54, 55, 59), as well as in countries facing obesity challenges like the US and Canada (60), NNS usage in beverages ranges from 20% to 40%, highlighting the persistence of this approach regardless of national nutrition policies. Further analysis based on THCL status revealed that beverages carrying the THCL logo were more likely to be classified as “healthier” due to their significantly lower energy and sugar content compared to unlabeled beverages. However, these products also showed a higher prevalence of NNS use, aligning with findings from previous studies on newly launched juice drinks in the Thai market (61). Given the high consumption of SSBs among Thai consumers (17), the increased use of NNS offers a potential strategy to reduce overall caloric intake from beverages. However, while NNS can lower calorie content, they do not entirely eliminate dietary sugar intake. Although the long-term health effects of NNS consumption remain inconclusive, concerns have been raised regarding their potential adverse effects (62). Despite the growing substitution of sugar with NNS, the widespread consumption of beverages means that overall sugar intake remains substantial (14, 63). This study provides a foundation for future research on consumer acceptance of NNS-containing beverages and their impact on dietary habits. Understanding the factors influencing NNS usage is essential for guiding public health strategies aimed at reducing excessive sugar intake. A comprehensive approach, incorporating collaboration between policymakers, the food industry, and health professionals, is necessary to implement evidence-based interventions that promote healthier beverage choices and improve public health outcomes (16, 63).

### How does the nutritional composition of pre-packaged beverages in Thailand influence their healthfulness profile?

Building on the findings about sugar content and sweetener use, we then evaluated the healthfulness profile of these beverages to assess their overall nutritional quality. Although the beverages analyzed in our study had lower sugar content, the majority (81.6%) were still categorized as “less healthy,” falling into grades “C,” “D,” and “E” according to the Nutri-Score algorithm. These findings are consistent with studies conducted in several EU countries, such as Greece and Germany with proportion of unhealthy beverages was 84.9% and 90.0%, respectively (64, 65), and align with a previous analysis of newly launched products in the Thai market from 2015 to 2021, where 72.4% of beverages were classified in the marketing-prohibited group based on the WHO SEA model (66). Using the HSR scheme, our study found that the mean ± standard deviation HSR score was 1.64 ± 0.8. This suggests that the overall nutritional profile of pre-packaged beverages in Thai supermarkets is comparable to those in emerging countries such as China, India, Mexico, Chile, and South Africa, where mean HSRs range from 1.6 to < 2.5 (53). In contrast, developed countries like the US, Australia, New Zealand, and the United Kingdom tend to have healthier beverage profiles, with mean HSRs of 2.5 or higher (53, 67). This observation aligns with a recent global report comparing the nutritional quality of foods sold by the world's largest food and beverage manufacturers, which found that products available in higher-income countries generally had higher mean HSRs than those in middle-income countries (68).

Several factors contribute to these disparities. First, differences in food labeling regulations, as well as the implementation and enforcement of labeling policies, influence the overall healthfulness of products available in high- and middle-income countries (69). Second, national variations in product portfolios, including the types and compositions of beverages in different markets, play a significant role. For example, in Australia and New Zealand, <10% of SSBs contain NNS (70), whereas in Thailand, this proportion is as high as 40%. Since NNS are commonly used to replace added sugars, their presence is typically associated with a lower mean total sugar density in beverages (71). Third, differences in how NPSs classify beverages and account for NNS may weaken the correlation between healthfulness ratings and total sugar content. For instance, the HSR and CWL do not factor NNS into their scoring algorithms, whereas Nutri-Score does (36, 49, 50). Additionally, while the THCL and Nutri-Score schemes classify MCC drinks and plant-based substitutes as beverages (22, 48), the HSR system categorizes these products differently, as either beverages (Group 1) or dairy products (Group 1D), depending on their calcium content (36). These discrepancies can lead to inconsistencies in healthfulness assessments, as beverages with NNS or different categorical classifications may appear healthier under some systems but not others. The algorithm of Nutri-Score and HSR may not provide strong incentives for soft drink manufacturers to reformulate their products. For instance, under HSR, beverages with any amount of sugar above 0 g automatically receive a 3-star rating, while Nutri-Score assigns them a Grade C, even if the sugar content is as low as 0.1 g. These ratings remain unchanged until sugar content exceeds 3.5 g per 100 ml, meaning that manufacturers are not rewarded for gradual sugar reductions below this threshold. Unlike food products, soft drinks do not benefit from incremental improvements in ratings for further sugar reduction, limiting the ability of these schemes to encourage reformulation or effectively guide consumers toward healthier choices (65, 70). Given these challenges, countries with diverse food supplies may need to adjust NPS cut-off points to ensure more accurate health assessments of beverages. A more tailored approach to scoring could improve the effectiveness of labeling systems in promoting healthier choices (40, 66). Ultimately, revising these systems, while incorporating incentives for reformulation, can enhance the nutritional quality of the food supply, support healthier dietary patterns, and contribute to improved public health outcomes (69).

### Do the sugar levels in pre-packaged beverages meet WHO “free sugar” recommendations?

Following the analysis of sugar levels in beverages, we assessed their compliance with WHO sugar consumption guidelines (16). Our findings indicate that the median serving size specified by manufacturers for the pre-packaged beverages examined was 200.0 ml (Supplementary Table 4). However, in Thailand, beverages are frequently sold in larger containers of 500 or 600 ml, which are often consumed in a single sitting despite nutrition labels indicating that these bottles contain two or three servings. As a result, regardless of whether a beverage contains only NNS or a combination of NS and NNS, consuming an entire bottle would easily exceed the WHO's stricter guideline of limiting free sugar intake to <5% of total daily energy. Notably, carbonated drinks and tea are the most commonly consumed beverages among Thai children and adults, with their typical portion and servings sizes of 500 and 250 ml, respectively (72, 73). When consumed in one sitting, these beverages may contribute to more than 10% of an individual's daily energy intake. These findings align with a recent study conducted in Singapore and Australia (55). As global trends shift toward increased consumption of beverages containing either exclusively NNS or a combination of NS and NNS (25), these results underscore the need for public health interventions that address portion sizes and sugar content. Even a single serving of these beverages can significantly contribute to exceeding recommended free sugar limits, particularly when consumed in larger quantities. Given the ongoing concerns about excessive sugar intake, continuous monitoring and assessment of evolving sweetener usage trends are essential (25). This assessment not only highlights the substantial role of beverages in dietary sugar consumption but also reinforces the need for reformulation efforts to reduce sugar content and promote healthier beverage options (74).

### Which sugar criteria in current front-of-package nutrition labeling (FOPNL) schemes should be revised to enhance beverage healthfulness and ensure alignment with WHO “free sugar” recommendations?

Given our findings indicating the “less healthy” profile of pre-packaged beverages in Thai supermarkets and the concern that they easily exceed the WHO “Free Sugar” recommendations, it is crucial to improve their healthfulness by reconsidering the total sugar criteria used in the FOPNL, particularly THCL scheme. Our study suggested a phased approach to lowering sugar benchmarks in the THCL criteria, first reducing the threshold to 5.0 g/100 ml, followed by a further reduction to 4.0 g/100 ml. The initial reduction to 5.0 g/100 ml aligns with existing NPSs that define healthier beverage options, such as the threshold T1 and T2 of the Choices Five-level scheme (75), Grade B in Nutri-Grade and Healthier Choice labels in Singapore (76, 77), and warning label schemes in Israel, Chile, Brazil, Mexico, and Peru (50, 78–81). However, the AUC for this threshold did not demonstrate significantly greater discriminatory power compared to the existing 6.0 g/100 ml threshold. In contrast, when the threshold is further reduced to 4.0 g/100 ml, the AUC values reach their highest levels, suggesting improved alignment with established NPSs. Notably, this threshold exhibits strong comparability with Nutri-Score scheme, which has substantial criterion validation evidence, and the HSR system, which has intermediate validation evidence (40). These findings were also corroborate and reinforce our previous research involving a large database of nearly 24,000 sugary beverages from Open Food Facts (82). The adoption of a 4.0 g/100 ml threshold offers several advantages. First, it improves alignment with the WHO “Free Sugar” recommendations. Previous research indicated that beverage manufacturers frequently reformulate products to stay just below or equal to FOPNL sugar thresholds to achieve positive labeling or avoid negative labeling. Evidence of this behavior includes product clustering at specific cut-off points, as observed in systems like Nutri-Score (83). Given that beverages in Thailand are typically sold in 500–600 ml containers, the consumption of a product containing 5.0 g/100 ml of sugar may result in total sugar intake exceeding 25 g/day, which is equivalent to 5% of total daily energy intake in one-sitting. A stricter threshold of 4.0 g/100 ml may therefore provide stronger consumer protection by reducing the likelihood of excessive sugar intake. Furthermore, this threshold represents a balanced approach between more stringent limits, such as the 2.5 g/100 ml criterion used to define “low sugar” beverages in the United Kingdom and the EU (84), and more lenient thresholds, such as the current criteria of 6.0 g/100 ml. This intermediate threshold is likely to facilitate industry adaptation while supporting public health objectives. Given the increasing consumer demand for healthier products, tightening the sugar threshold in the THCL system has the potential to incentivize manufacturers to reformulate products, thereby improving the overall nutritional quality of the food supply while maintaining consistency with existing nutrition labeling policies.

While implementing a stricter sugar threshold offers potential health benefits, several key factors must be carefully considered to ensure its effectiveness. First, as carbohydrates encompass various nutrients, the treatment of fiber and naturally occurring sugars, such as those present in FVNL in Nutri-Score and FVPNRWO in HSR, varies across different NPSs (36, 49). Additionally, the inclusion or exclusion of NNS in NPS scoring frameworks influences both sweetness perception and the appropriate sugar threshold (22, 50). Second, a key consideration is whether a uniform sugar threshold should be applied across all beverage types or tailored to specific product categories. For instance, the Choices Five-Level scheme establishes distinct sugar thresholds for fruit and vegetable juices, non-dairy substitutes, and other beverages, ensuring category-specific assessments (75). Third, differences in the design and interpretability of FOPNL systems impact both consumer understanding and industry response. Nutri-Score and HSR use multi-tiered, graded systems that allow consumers to compare healthfulness across and within food and beverage categories. These systems can encourage manufacturers to make incremental product improvements by offering better ratings for progressively healthier formulations (36, 48). In contrast, the THCL operates as a binary system, where products either meet the criteria and receive the logo or fail to qualify (22). While this pass/fail approach offers clarity, it lacks the nuanced classification found in graded systems, meaning that products cannot be ranked as “moderately healthy”, they are either classified as meeting the standard or not (85). Another critical distinction lies in how these systems evaluate overall nutritional quality. Nutri-Score and HSR allow for compensation, meaning that a beverage high in sugar but also high in fiber may still receive a mid-range score (36, 49). In contrast, THCL establishes predefined nutrient limits per category, emphasizing compliance with minimum standards. This strict threshold approach, while effective in setting clear limits, may lead to unintended consequences, such as manufacturers replacing sugar with artificial sweeteners without improving the product's overall nutritional quality. Additionally, threshold-driven reformulation, where manufacturers reduce sugar just enough to meet the cut-off, may not lead to meaningful long-term improvements in food and beverage healthfulness (23). Therefore, when revising the sugar threshold in the THCL scheme, it is essential to balance public health objectives with industry feasibility. Policymakers should carefully weigh the trade-offs between simplicity and comprehensiveness when selecting or adapting FOPNL systems (84). An appropriately designed threshold should encourage substantial reductions in sugar content, support gradual reformulation by the food industry, and provide consumers with clear, effective labeling. By considering these factors, policymakers can develop an evidence-based revision to the THCL system that strengthens public health efforts to combat obesity and NCDs while promoting sustainable food production and reformulation initiatives.

This study has several limitations that should be acknowledged. Firstly, our analysis centered exclusively on pre-packaged beverages available in supermarkets within Bangkok. While this approach allowed us to leverage the most comprehensive nutritional composition data currently accessible from Thai food retailers, it's important to acknowledge that the findings might not fully represent the diverse range of beverage products present in the nationwide Thai food supply. Nevertheless, supermarkets play a significant role in shaping dietary habits due to their influence on food accessibility, making their available products highly relevant to public health and nutrition considerations. Secondly, flavored milk beverages were excluded from our analysis. This decision was based on their classification as dairy products by the Thai FDA and their distinct categorization under the HSR scheme, which considers calcium content criteria that differ significantly from those of plant-based milk alternatives included in our study (33, 34, 36). Thirdly, our database did not encompass the sugar and/or sugar substitutes present in freshly prepared beverages sold at popular outlets such as All Coffee^®^, Amazon^®^, and Starbucks™. The study specifically focused on pre-packaged beverages in Thai supermarkets, where mandatory sugar labeling provides a reliable data source, unlike freshly prepared beverages that often lack standardized nutritional labeling. Consequently, our findings may not capture the complete spectrum of sugar consumption from all beverage sources available to Thai consumers. Finally, it should be noted that information regarding FVNL or FVPNRWO content is not currently mandatory on the standard NIP in Thailand. Therefore, any estimations of these values may introduce minor inaccuracies. Despite these limitations, this study offers valuable insights into the sugar content of pre-packaged beverages in Bangkok supermarkets, contributing to a broader understanding of Thai consumers' dietary composition. These findings can provide critical guidance for policymakers and researchers in formulating informed sugar reduction strategies and directing future research efforts in this important area.

## Conclusion

In conclusion, this study highlights the “less healthy” nutritional profile and high prevalence of NNS in pre-packaged beverages sold in Thai supermarkets. Products labeled with the THCL logo generally exhibited a “healthier” profile, largely due to their higher use of NNS compared to unlabeled beverages. However, regardless of whether beverages contain only NS or a mix of NS and NNS, consuming a single 500- or 600-ml bottle easily exceeds the WHO's stricter recommended limit of 5% of total daily energy intake from free sugars. This finding underscores the urgent need to reduce sugar content in beverages. To achieve a “win-win” solution that prioritizes public health while considering the practical challenges faced by the food and beverage industry, this study suggests revising the THCL scheme by implementing incremental sugar benchmarks. Specifically, the sugar threshold should first be lowered to 5.0 g/100 ml, followed by a further reduction to 4.0 g/100 ml. These adjustments would improve the THCL scheme's ability to differentiate the healthfulness of beverages and ensure better alignment with WHO guidelines on sugar limits. Ultimately, such revisions could enhance the overall nutritional quality of beverages available in Thailand and support broader efforts to promote healthier dietary choices.

## Data Availability

The raw data supporting the conclusions of this article will be made available by the authors, without undue reservation.
