# Supplementary material for: Assessing the healthfulness of pre-packaged beverages and investigating sugar thresholds for revising front-of-pack labels: a case study in Thailand
Source: Front Nutr. 2025 Jun 2;12:1564216. doi: 10.3389/fnut.2025.1564216 (PMC12168469; doi:10.3389/fnut.2025.1564216)
Supplement: Supplementary file 1 [file Table_1.docx]

Supplementary Material

**Assessing the healthfulness of pre-packaged beverages and investigating sugar thresholds for revising front-of-pack labels: A case study in Thailand**

**Hung Nguyen Ngoc^1^, Mayuree Ditmetharoj^2^, Nipa Rojroongwasinkul^3^, Pattanee Winichagoon^3^, Wantanee Kriengsinyos^3^**

^1^ Doctor of Philosophy Program in Nutrition, Faculty of Medicine Ramathibodi Hospital and Institute of Nutrition, Mahidol University, Nakhon Pathom 73170, Thailand

^2^ Food and Drug Administration, Ministry of Public Health, Nonthaburi 11000, Thailand

^3^ Food and Nutrition Academic and Research Cluster, Institute of Nutrition, Mahidol University, Salaya, Phutthamonthon, Nakhon Pathom 73170, Thailand

*** Correspondence:** Wantanee Kriengsinyos, wantanee.krieng@mahidol.ac.th

**Table of Contents**

[**1. Supplementary Figure 1.** Steps to Estimate FVPNRWO and FVNL Content (1) 3](#_Toc192076831)

[**2. Supplementary Table 1.** List, definitions and product examples of categories of pre-packaged beverages adapted from the Codex Alimentarius food category system 4](#_Toc192076832)

[**3. Supplementary Table 2.** Definitions of sweeteners used in this study 6](#_Toc192076833)

[**4. Supplementary Table 3.** Calculating, grading and classifying process of different nutrient profiling systems (NPS) 7](#_Toc192076834)

[**5. Supplementary Table 4:** Sugar per serve and serve sizes for different pre-packaged beverage categories 9](#_Toc192076835)

[**6. Supplementary Table 5.** Area under the receiver operating characteristic curve (ROC-AUC) to discriminate between ‘healthier’ and ‘less healthy’ pre-packaged beverages used in the current total sugar threshold of THCL criteria and different sugar threshold scenarios 10](#_Toc192076836)

[References 11](#_Toc192076837)

# 1. Supplementary Figure 1. Steps to Estimate FVPNRWO and FVNL Content (1)


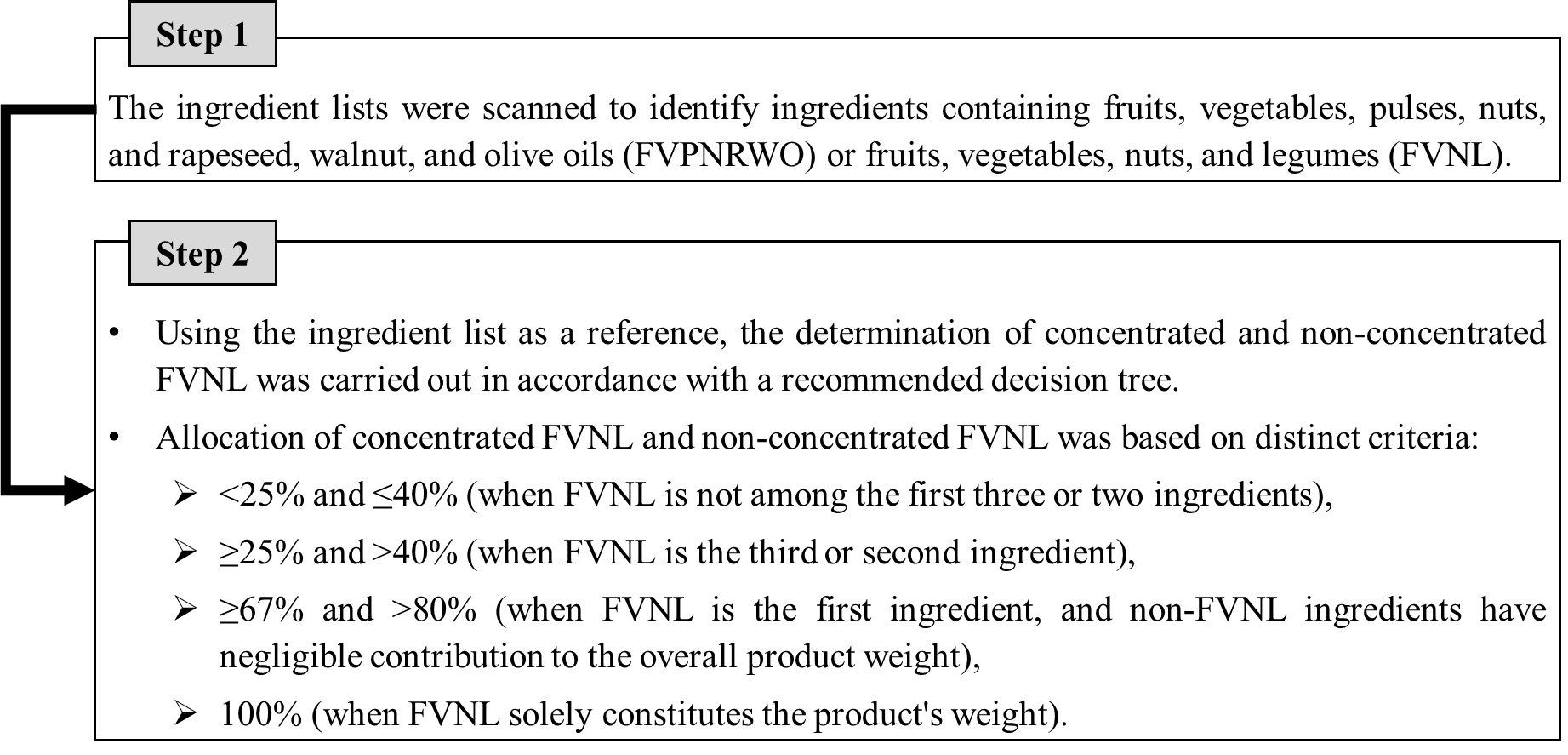


# 2. Supplementary Table 1. List, definitions and product examples of categories of pre-packaged beverages adapted from the Codex Alimentarius food category system

| **No.** | **Sub-category** | **Definition** |
| --- | --- | --- |
| 1 | Carbonated drink | This includes all carbonated beverages that contain some form of sweetener/additive or flavoring except for tea products. It comprises various such drinks like creaming sodas, lemonades, lemon, lime, bitters, sarsaparilla, other flavored soft drinks, frozen soft drinks of any type, tonic water, club soda, flavored sparkling waters. |
| 2 | Coffee | This category includes coffee-based beverages where milk is not the primary ingredient. It encompasses both ready-to-drink coffee and instant coffee, which can be either carbonated or non-carbonated. These products are available in various formats, such as powdered, ground, or packed in bags for preparation, as well as pre-packaged in liquid forms like cans or bottles for immediate consumption. |
| 3 | Energy drink | This category captures both carbonated and non-carbonated beverages that are marketed as "energy enhancers." |
| 4 | Flavored water | This category covers flavored non-carbonated beverages that do not fit into other classifications. It includes cordial concentrates, ready-to-drink cordials, syrups, herbal drinks, coconut water, fruit-flavored drinks, and still water with added flavorings. |
| 5 | Malted, chocolate, and cocoa drinks (abbreviated as MCC drinks) | These beverages primarily contain chocolate and/or cocoa as their main ingredient or predominant flavor. This category includes powdered, ground, or ready-to-drink malted beverages, commonly known as chocolate malted drinks. |
| 6 | Non-100% fruit and vegetable juice drink (abbreviated as Non-100% FVJ drink) | This category includes beverages that contain less than 100% fruit or vegetable juice, typically made through the extraction or pressing of natural liquids from fruits and vegetables. These drinks often combine juice with other ingredients, such as water, sweeteners, or flavorings, resulting in a diluted juice product. |
| 7 | Plant-based milk substitute | This category includes all beverages defined as non-dairy, which are mainly liquid concentrates based on different plant raw materials such as cereals and rice, peas and soy in addition to different seeds and nuts. |
| 8 | Sports drink | This category covers non-carbonated drinks marketed as "sports enhancers" frequently including added vitamins and minerals. |
| 9 | Tea | This category includes tea where milk is not the primary ingredient. It covers both ready-to-drink tea and instant tea, which are primarily made from black or green tea and can be consumed either hot or cold. These beverages may be carbonated or non-carbonated and are available in a variety of formats, such as powdered, ground, tea bags, or pre-packaged ready-to-drink options like bottled or canned products. |

# 3. Supplementary Table 2. Definitions of sweeteners used in this study

| Nutritive Sweetener (NS) | Nutritive sweeteners are sugars and caloric sweeteners that occur naturally (intrinsic) in fruits, vegetables, and dairy products or are added (extrinsic) to foods during processing or preparation. This category encompasses a wide variety of sweeteners, including sugar itself and other caloric sweeteners such as fruit juice concentrate (FJC), cane sugar, beet sugar, sucrose, corn syrup, high-fructose corn syrup (HFCS), agave-based sweeteners, honey, molasses, maple syrup, sorghum, malt, maltose, rice syrup, fructose, lactose, and inverted sugars. Additionally, it also includes sugar alcohols like sorbitol (E420/INS420), mannitol (E421/INS421), isomalt (E953/INS953), polyglycitol syrup (E964/INS964), maltitol (E965/INS965), lactitol (E966/INS966), xylitol (E967/INS967), and erythritol (E968/INS968), as well as low-calorie sweeteners and other caloric sweeteners such as gomme and starch-based sweeteners. |
| --- | --- |
| Non-nutritive Sweetener (NNS) | Non-nutritive sweeteners include no-calorie sweeteners, both artificial and natural, that serve as alternatives to sugars in a variety of foods and beverages. The list of non-nutritive sweeteners used in this study is adapted from the 2019 Codex Alimentarius General Standard for Food Additives (2) and Regulation (EC) No 1333/2008 of the European Parliament and Council on food additives (3). The list below details the specific sweeteners including, Acesulfame K (E950/ INS950), Aspartame (E951/ INS951), Cyclamates (E952/ INS952 (IV), Saccharin (E954/ INS954), Sucralose (E955/ INS955), Alitame (E956/ INS956), Thaumatin (E957/ INS957), Neohesperidine DC (E959/ INS959), Steviol glycosides from Stevia (E960a/ INS960), Neotame (E961/ INS961), Salt of aspartame-acesulfame (E962/ INS962), Advantame (E 969/ INS 969) |

# 4. Supplementary Table 3. Calculating, grading and classifying process of different nutrient profiling systems (NPS)

|  | **Nutri-Score** | | **Health Star Rating (HSR)** | | | **Chilean Warning Label (CWL)** | |
| --- | --- | --- | --- | --- | --- | --- | --- |
| **Introduction** | Nutri-Score is a front-of-pack nutrition labeling system that uses five letters (A, B, C, D, and E) to indicate a product's nutritional quality, with each letter associated with a specific color. This system is widely implemented in European countries (4,5). | | Health Star Rating system is a voluntary interpretive front-of-pack nutrition labeling scheme implemented in Australia and New Zealand. This system employs a rating scale ranging from 0.5 to 5.0 stars, with half star increments, wherein a higher number of stars signifies a healthier product (6). | | | The Chilean Warning Label, introduced in 2016, features black-and-white octagons. Its purpose is to inform consumers about the nutritional quality of packaged foods and promote healthier eating habits. (7). | |
| **Version** | The updated Nutri-Score algorithms (V2.2023) for beverages were utilized, following guidelines from Santé Publique France (4,5). | | The HSR calculation for all pre-packaged beverages in this study adhered to the methodology outlined in the 'Guide for Industry to the Health Star Rating Calculator' (V8. 2023) (6). | | | The warning labeling system uses an algorithm to determine if products exceed nutrient thresholds established by the Chilean Ministry of Health. The thresholds applied are those that were in effect as of June 27, 2019. (7) | |
| **Calculating** | - The modified Food Standard Agency Nutrient Profiling System (FSAm-NPS) score based on the nutritional content per 100ml of each beverage. - This analysis involved subtracting the score for "negative nutrients" (energy, total sugars, saturated fat, sodium, and the presence of non-nutritive sweeteners) from the score for "positive nutrients" (fruits, vegetables, pulses, nuts, and specific oils content), with higher scores for greater nutrient content. - The resulting FSAm-NPS scores ranged from -15 (healthiest) to +40 (least healthy). | | - Most beverages were generally classified under Group 1, except for plant-based milk substitutes, which could belong to Group 1D based on their calcium content (at least 100mg calcium per 100ml). - The HSR score was calculated by deducting 'modifying' points from 'baseline' points. - For beverages, baseline points were determined based on energy and total sugar content per 100 mL, while modifying points were assigned for the fruit, vegetable, nut, and legume content (FVNL%). - Subsequently, this score was converted into a Health Star Rating using a predefined scoring matrix. | | | - The warnings are based on nutritional data from 100 mL of beverages. If a product exceeds the acceptable limits for sugar, sodium, saturated fat, or calories set by the Chilean Ministry of Health, the label will display the phrase “High in.” - Each product must display a warning for each nutrient that surpasses these thresholds. Consequently, some products may have up to four labels, including: [1] High in energy; [2] High in sugar; [3] High in saturated fat (SATFAT); and [4] High in sodium.   The threshold for liquid products:   \| Energy \| >293kJ or 70kcal/100mL \| \| --- \| --- \| \| SATFAT \| >3g/100mL \| \| Sugar \| >5g/100mL \| \| Sodium \| >100mg/100mL \| | |
| **Grading** |  | **Group 1** |  | **Group 1** | **Group 1D** | **Color code** | **No. of warning label** |
|  | A | Water | 5 | Plain water | ≤ -2 |  | 0 |
|  | B | < 2 | 4.5 | Unsweetened flavored water | -1 |  | 1 |
|  | C | 3 – 6 | 4 | ≤0 | 0 |  | 2 |
|  | D | 7 – 9 | 3.5 | 1 | 1 |  | 3 |
|  | E | 10 – 40 | 3 | 2 – 3 | 2 |  | 4 |
|  |  | | 2.5 | 4 – 5 | 3 |  | |
|  |  |  | 2 | 6 – 7 | 4 |  |  |
|  |  |  | 1.5 | 8 – 9 | 5 |  |  |
|  |  |  | 1 | 10 – 11 | 6 |  |  |
|  |  |  | 0.5 | ≥ 12 | ≥ 7 |  |  |
| **Classifying** | 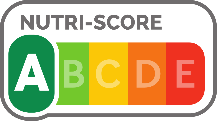  Classifying beverages, letters A and B represent products of high nutritional quality or 'healthier' options, while C denotes foods of balance choice. Conversely, letters D and E are used for products of low nutritional quality or 'less healthy' options (4,5). | | 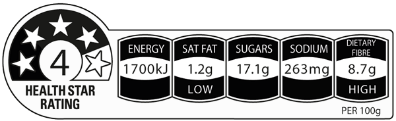  Classifying beverages, a higher HSR score indicates a healthier product. Beverages with an HSR of 3.5 or higher are considered "healthy," while those with an HSR below 3.5 are deemed "less healthy" and not aligned with healthy dietary guidelines (8). | | | 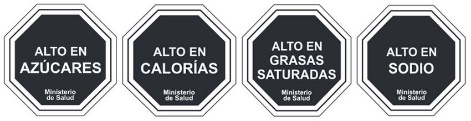  Beverages are classified based on their nutrient content. Those exceeding specific limits receive a warning label indicating they are "high in" particular nutrients. Consequently, a product can have no labels indicating it is "healthy," or up to four labels, indicating it is "less healthy." (7). | |

Abbreviations: FOPNL, Front-of-package nutritional labelling; FSAm-NPS, Modified Food Standard Agency nutrient profiling system; HSR, Health star rating; NPS, Nutrient profiling system; SATFAT, Saturated fat

# 5. Supplementary Table 4: Sugar per serve and serve sizes for different pre-packaged beverage categories

| **Beverage category** | **Only NS Beverage** | | | | **Mix NS + NNS Beverage** | | | |
| --- | --- | --- | --- | --- | --- | --- | --- | --- |
|  | **Sugar**^‡^ **per serve (g)** | **IQR** | **Serve size**^†^ **(mL)** | **IQR** | **Sugar**^‡^ **per serve (g)** | **IQR** | **Serve size**^†^ **(mL)** | **IQR** |
| Carbonated drinks | 18.0 | 14.3-21.5 | 225.0 | 200.0-250.0 | 15.0 | 12.0-15.0 | 250.0 | 200.0-325.0 |
| Coffee | 10.0 | 8.0-14.0 | 170.0 | 144.5-220.7 | 7.0 | 5.0-9.5 | 180.0 | 134.3-180.0 |
| Energy drinks | 28.0 | NA | 170.0 | NA | 10.5 | 8.3-17.8 | 150.0 | 145.0-150.0 |
| Flavored waters | 9.0 | 5.0-15.5 | 150.0 | 42.0-189.3 | 9.0 | 6.0-14.8 | 164.0 | 150.0-320.0 |
| MCC drinks | 15.0 | 12.0-17.0 | 218.0 | 185.0-230.0 | 12.0 | NA | 176.0 | NA |
| Non-100% FVJ drinks | 17.5 | 13.0-23.3 | 200.0 | 45.0-200.0 | 12.0 | 10.0-16.0 | 200.0 | 200.0-280.0 |
| Plant-based milk substitutes | 8.5 | 5.0-12.0 | 200.0 | 180.0-250.0 | 7.5 | 5.5-9.8 | 175.0 | 173.0-275.0 |
| Sports drinks | 13.0 | 12.0-26.8 | 225.0 | 200.0-250.0 | 15.5 | NA | 250.0 | NA |
| Tea | 16.0 | 4.5-20.0 | 250.0 | 200.0-300.0 | 13.5 | 7.8-17.0 | 250.0 | 190.0-350.0 |
| Total | 12.0 | 7.0-17.0 | 200 | 163.5-250.0 | 10.5 | 7.0-15.0 | 200.0 | 150.0-265.0 |

^†^Serving size was calculated as the median value of all products within that sub-category, based on the manufacturer-declared serving size information on the NIP

^‡^Calculated for sugar (either natural or added)-containing products only.

Abbreviation: IQR, Interquartile range; FVJ, Fruit Vegetable Juice; MCC, Malted Chocolate and Cocoa; NNS, Non-nutritive sweetener; NS, Nutritive Sweetener; N/A, not applicable

# 6. Supplementary Table 5. Area under the receiver operating characteristic curve (ROC-AUC) to discriminate between ‘healthier’ and ‘less healthy’ pre-packaged beverages used in the current total sugar threshold of THCL criteria and different sugar threshold scenarios

| **Sugar Threshold** | **6.0g/ 100mL** | **5.5g/ 100mL** | **5.0g/ 100mL** | **4.5g/ 100mL** | **4.0g/ 100mL** | **3.5g/ 100mL** | **3.0g/100mL** | **2.5g/ 100mL** | **2.0g/100mL** |
| --- | --- | --- | --- | --- | --- | --- | --- | --- | --- |
| **Nutri-Score** | | | | | | | | | |
| ROC-AUC | 0.702  (0.655-0.739) | 0.727  (0.691-0.764) | 0.745  (0.709-0.781) | 0.761  (0.723-0.799) | 0.768  (0.729-0.807) | 0.757  (0.714-0.799) | 0.753  (0.710-0.797) | 0.747  (0.702-0.792) | 0.733  (0.686-0.780) |
| SE | 0.019 | 0.019 | 0.018 | 0.019 | 0.020 | 0.020 | 0.022 | 0.023 | 0.024 |
| Sensitivity | 96.3  (92.1-98.6) | 94.4  (89.7-97.4) | 92.6  (87.4-96.1) | 85.8  (79.5-90.8) | 82.7  (76.0-88.2) | 75.9  (68.6-82.3) | 72.2  (64.7-79.0) | 68.5  (60.8-75.6) | 63.6  (55.7-71.0) |
| Specificity | 44.1  (40.4-47.8) | 51.0  (47.3-54.8) | 56.5  (52.8-60.1) | 66.3  (62.8-69.8) | 70.9  (67.5-74.2) | 75.4  (72.1-78.5) | 78.4  (75.3-81.4) | 81.0  (77.9-83.8) | 83.0  (80.1-85.7) |
| p-value | - | 0.466 | 0.206 | 0.080 | 0.049 ^sig^ | 0.103 | 0.131 | 0.185 | 0.364 |
| **Health Star Rating (HSR)** | | | | | | | | | |
| ROC-AUC | 0.685  (0.650-0.721) | 0.692  (0.657-0.728) | 0.698  (0.662-0.734) | 0.715  (0.678-0.751) | 0.722  (0.685-0.759) | 0.718  (0.681-0.755) | 0.717  (0.679-0.754) | 0.709  (0.671-0.748) | 0.693  (0.654-0.733) |
| SE | 0.018 | 0.018 | 0.018 | 0.019 | 0.019 | 0.019 | 0.019 | 0.020 | 0.020 |
| Sensitivity | 87.9  (83.6-91.4) | 82.8  (78.1-86.9) | 78.8  (73.7-83.3) | 71.7  (66.2-76.8) | 68.4  (62.7-73.6) | 63.0  (57.2-68.5) | 59.6  (53.8-65.2) | 55.9  (50.0-61.6) | 51.2  (45.3-57.0) |
| Specificity | 49.1  (45.0-53.3) | 55.7  (51.5-59.7) | 60.8  (56.7-64.8) | 71.2  (67.4-74.9) | 76.0  (72.4-79.4) | 80.7  (77.2-83.8) | 83.7  (80.5-86.6) | 86.0  (82.9-88.7) | 87.5  (84.5-90.1) |
| p-value | - | 0.800 | 0.637 | 0.273 | 0.175 | 0.227 | 0.242 | 0.382 | 0.772 |
| **Chilean Warning Label (CWL)** | | | | | | | | | |
| ROC-AUC | 0.871  (0.846-0.897) | 0.932  (0.913-0.951) | 0.980  (0.970-0.991) | 0.900  (0.877-0.923) | 0.862  (0.836-0.888) | 0.820  (0.791-0.850) | 0.789  (0.758-0.820) | 0.764  (0.732-0.797) | 0.741  (0.708-0.774) |
| SE | 0.013 | 0.010 | 0.005 | 0.012 | 0.013 | 0.015 | 0.016 | 0.016 | 0.017 |
| Sensitivity | 100.0  (99.2-100.0) | 100.0  (99.2-100.0) | 96.3  (94.2-97.9) | 82.7  (78.9-86.1) | 74.7  (70.4-78.6) | 65.7  (61.1-70.1) | 59.4  (54.7-64.0) | 54.3  (49.5-59.0) | 49.3  (44.6-54.1) |
| Specificity | 74.3  (69.9-78.3) | 86.4  (82.9-89.5) | 100.0  (99.1-100.0) | 97.2  (95.2-98.6) | 97.7  (95.8-98.9) | 98.4  (96.7-99.4) | 98.4  (96.7-99.4) | 98.6  (97.0-99.5) | 98.9  (97.3-99.6) |
| p-value | - | <0.001 ^sig^ | <0.001 ^sig^ | 0.07 | 0.615 | 0.006 ^sig^ | <0.001 ^sig^ | <0.001 ^sig^ | <0.001 ^sig^ |

Note: The value was expressed as mean with 95% confidence interval (CI) with the sensitivity and specificity was calculated as equated below:

$Sensitivity=\frac{True positive}{True positive + False negative}$; $Specificity=\frac{True negative}{False positive + True negative}$;

The identification of 'healthier' sugar-sweetened beverages according to various nutrient classification schemes was determined as follows: (a) Nutri-Score: Grades A and B; (b) Health Star Rating: HSR ≥ 3.5 stars; (c) Chilean Warning Label: Products that do not meet any warning criteria. The 'less healthy' were assigned to the remaining grades. The P-value indicates the significant differences between the AUC of current sugar threshold scenarios (6g/ 100mL) and other sugar scenarios, sig indicates the significant differences at p-value <0.05. Abbreviation: ROC-AUC, Area under the receiver operating characteristic curve; CI, Confidence interval; SE: Standard error.

# References

1. Vergeer L, Ahmed M, Franco-Arellano B, Mulligan C, Dickinson K, Bernstein JT, Labonté M-È, L’Abbé MR. Methodology for the Determination of Fruit, Vegetable, Nut and Legume Points for Food Supplies without Quantitative Ingredient Declarations and Its Application to a Large Canadian Packaged Food and Beverage Database. *Foods* (2020) 9:1127. doi: 10.3390/foods9081127

2. FAO, WHO. Codex Alimentarius, General Standard For Food Additives. (2019) https://www.fao.org/gsfaonline/docs/CXS_192e.pdf

3. Regulation - 1333/2008 - EN - additives - EUR-Lex. https://eur-lex.europa.eu/eli/reg/2008/1333/oj/eng [Accessed March 1, 2025]

4. Santé Publique France. Conditions of Use of the “Nutri-Score” Logo. (2023)

5. Santé Publique France. Update of the Nutri-Score algorithm for beverages: Second update report from the Scientific Committee of the Nutri-Score V2 – 2023. https://sante.gouv.fr/IMG/pdf/update_report_beverages_31_01_2023-voted.pdf

6. Australian Department of Health. Health Star Rating system: Calculator and Style Guide: October 2023, Version 8. (2023) http://www.healthstarrating.gov.au/internet/healthstarrating/publishing.nsf/Content/E380CCCA07E1E42FCA257DA500196044/$File/HSR%20System%20Calculator%20and%20Style%20Guide%20v8.pdf

7. Reyes M, Garmendia ML, Olivares S, Aqueveque C, Zacarías I, Corvalán C. Development of the Chilean front-of-package food warning label. *BMC Public Health* (2019) 19:906. doi: 10.1186/s12889-019-7118-1

8. Dunford E., Cobcroft M., Thomas M., Wu J. M. Technical Report: Alignment of NSW Healthy Food Provision Policy with the Health Star Rating System. *New South Wales Ministry of Health; Sydney, Australia: 2015*88.
